# Flexible Wireless Passive Resonance Ring Sensor for Nondestructive Crack Monitoring of Metal Structures

**DOI:** 10.3390/mi17080943

**Published:** 2026-08-07

**Authors:** Yingmin Wang, Xiaodong Huang, Pan Pei

**Affiliations:** 1 Department of Intelligent and Information Engineering, Taiyuan University, Taiyuan 030032, China; 2024502001@tyu.edu.cn; 2 Key Laboratory of Instrumentation Science and Dynamic Measurement, North University of China, Taiyuan 030051, China; peipan@nuc.edu.cn

**Keywords:** resonant ring, crack detection, wireless passive, flexible sensor, sensitivity

## Abstract

Despite the aim of meeting the demand for long-term online monitoring of structural cracks in fields such as infrastructure, rail transit, aerospace and others, traditional detection methods fail to realize passive wireless, flexible conformal and non-contact measurement. This paper proposes a flexible wireless passive crack sensor based on resonant rings. Taking polyimide (PI) as the substrate, the sensor integrates a sensitive interdigital resonant ring structure. Variations in crack width disturb the electromagnetic field, which further leads to a resonant frequency shift to realize crack width detection. The sensing mechanism is elaborated based on microwave resonance and equivalent circuit theories. Structural optimization and crack width sensitivity analysis are carried out via electromagnetic simulation. Samples are fabricated by flexible printing technology, and a test platform is established. Experiments reveal that the sensor achieves excellent linearity within the crack width range of 0~2.5 mm, with the resonant frequency decreasing monotonically as crack width increases, and a sensitivity of 67.02 MHz/mm. It can operate stably under varying distances, installation angles and bending conditions, demonstrating outstanding flexible conformability. Featuring no power supply requirement, a chip-free design, a simple structure and strong anti-interference capability, the sensor is suitable for long-term crack monitoring of metal structures. Compared with existing studies, the proposed sensor exhibits prominent advantages in flexible adaptability, wireless passive performance and engineering practicability and can provide a novel wireless passive solution for structural health monitoring.

## 1. Introduction

Structural health monitoring is a key technology for ensuring the safe operation of modern engineering equipment, extending service life and reducing operation and maintenance risks, among which crack monitoring serves as the most core and fundamental link. Infrastructures such as bridges, tunnels and high-rise buildings, as well as high-end equipment including aircraft, high-speed trains, wind turbine blades and high-pressure pipelines, are prone to developing initial microcracks under long-term cyclic loads, impact vibrations, temperature fluctuations and corrosive environments. These microcracks will continuously propagate under sustained external forces, eventually leading to structural fracture, damage and even catastrophic accidents [1,2,3,4]. Accordingly, realizing early identification, real-time monitoring, quantitative measurement and trend prediction of cracks bears irreplaceable significance for safeguarding public safety and enhancing equipment reliability. Traditional crack monitoring methods mainly consist of visual inspection, ultrasonic testing, eddy current testing, X-ray testing, piezoelectric sensor testing and strain gauge measurement [5,6,7,8]. Although these methods are applicable to certain specific scenarios, they generally have prominent drawbacks: ultrasonic and eddy current tests require close-contact scanning and cannot support remote wireless monitoring; piezoelectric and strain sensors usually rely on wired power supply and signal transmission, which are prone to aging and damage in harsh environments with complicated wiring and are difficult to deploy in large-scale deployment; conventional nondestructive testing devices feature large volumes, high costs and complicated operation, failing to meet the demands of long-term, distributed and unattended monitoring [9,10,11,12,13,14]. Under special working conditions such as curved structures, rotating components, sealed cavities, high temperatures and high pressures, traditional sensors face greater challenges in conformal installation and stable operation.

Wireless passive sensors have become a research hotspot in the field of structural health monitoring in recent years due to their advantages such as battery-free operation, no wiring requirements, a long service life and convenient deployment. Microwave resonant sensors adopt resonant structures including resonant rings, split-ring resonators and complementary split-ring resonators as sensitive units and realize quantitative detection of crack widths through resonant frequency shifts induced by cracks, featuring high precision, fast responses and strong anti-interference capability. Ou et al. [15] proposed a microwave resonance-based detection method to measure the depth of hidden internal cracks in rocks and cement mortar, offering a new approach for internal defect detection of non-metallic materials. Chaudhary et al. [16] introduced Hilbert curve structures into microwave sensors, improving the resolution of defect detection for carbon fiber-reinforced composites. Cai et al. [17] designed reconfigurable wireless passive sensors to achieve high-sensitivity crack detection, verifying the feasibility of wireless passive structures for crack monitoring. Bait Suuwailam et al. [18] utilized multi-channel complementary split-ring resonators to detect cracks in concrete blocks, expanding the application scope of microwave sensors in the field of building structures. Zhang et al. [19] put forward a non-resonant dual-channel microwave sensor, which effectively mitigates environmental interference via dual-frequency decoupling and enhances the stability of crack detection. Kotriwar et al. [20] designed chipless hybrid RFID sensors to realize wireless detection of surface cracks on metals. Wang et al. carried out the design of passive RFID sensors for metal crack width measurement, validating the potential of passive schemes in practical engineering scenarios. Chu et al. [21] constructed quasi-distributed crack sensors based on weakly coupled vertical U-shaped arrays, enabling multi-location monitoring. Liu et al. [22] proposed a chipless RFID sensor capable of high-sensitivity detection of metal crack widths. Wang [23] and Wang [24] further investigated passive RFID crack sensors for metal surfaces, improving the applicability of the system in practical engineering. Although remarkable progress has been achieved in existing research, most sensors still suffer from multiple drawbacks including predominant rigid substrates, poor flexibility adaptability, difficulty in conformal installation, a short reading distance, severe susceptibility to environmental factors and a narrow linear monitoring range.

Based on the microwave resonance theory, this paper constructs an integrated flexible structure combining resonant rings and waveguide antennas and establishes a comprehensive system covering its working principles, equivalent circuit models, simulation procedures, fabrication processes and testing methods. The key research focuses on the influence of crack width on resonant frequency, the effects of testing distance and transmission angle on output characteristics, structural stability under flexible bending conditions, as well as the optimization of the linearity and sensitivity of the sensor. The research outcomes of this paper can be applied to long-term online crack monitoring in fields such as bridges, rail transit, aerospace, new energy equipment and pressure pipelines and possess significant theoretical value and engineering significance for advancing the practical application of wireless passive flexible sensing technology.

## 2. Working Principle of Wireless Passive Crack Sensor

**Figure 1:**
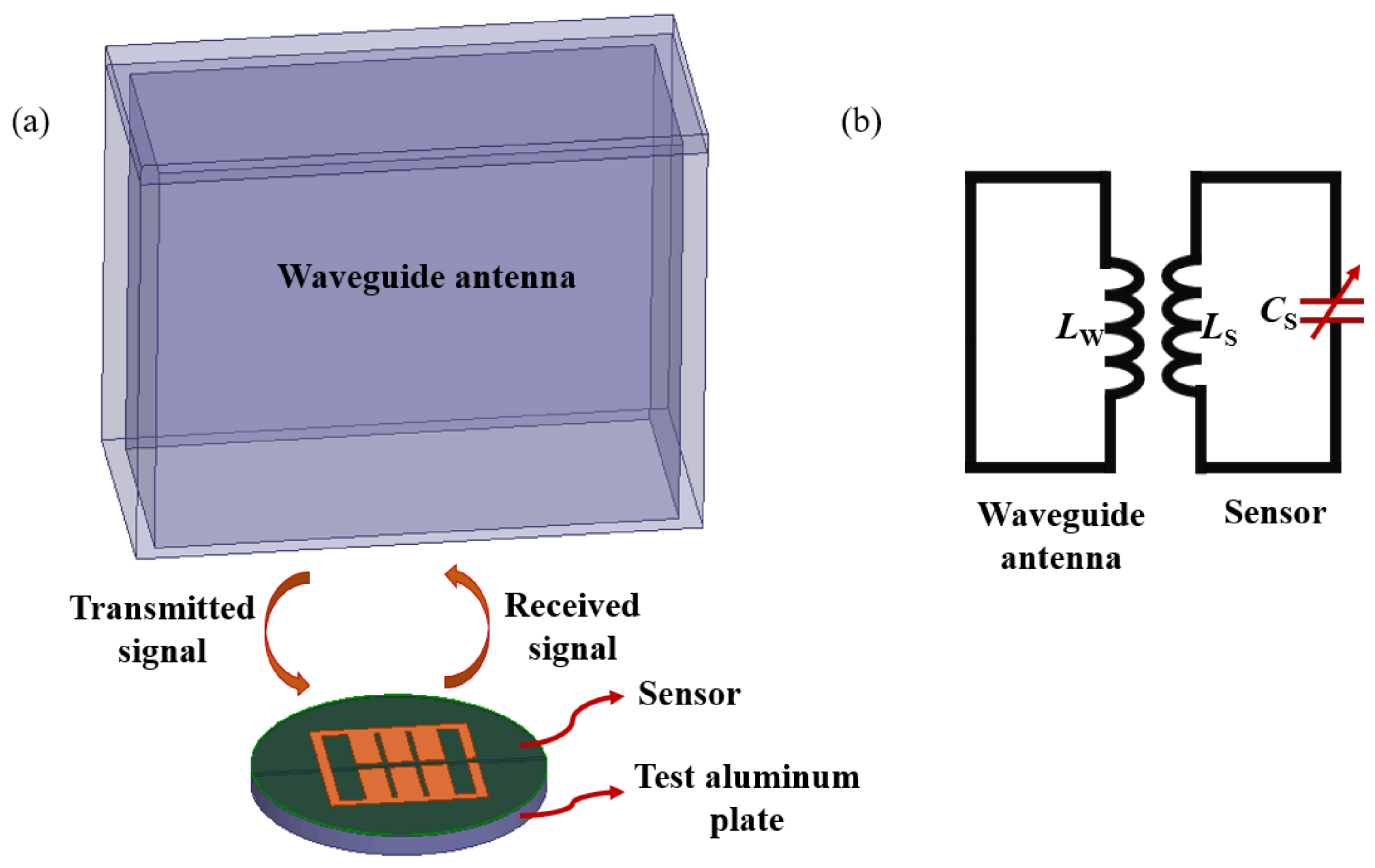
Working principle of the wireless passive crack sensor. (**a**) Wireless transmission principle of the wireless passive crack sensor. (**b**) Equivalent circuit diagram of the wireless passive crack sensor.

The flexible wireless passive crack sensor designed in this paper takes a microwave resonant ring as the core sensitive structure and cooperates with a waveguide antenna (WR-340) to realize the transmission, coupling and reception of microwave signals. Its overall working mechanism can be systematically elaborated from four perspectives: a wireless transmission principle, a resonance response mechanism, a sensitive modulation mode of crack width and an equivalent circuit model. The sensor is mainly composed of three parts: a flexible substrate, a resonant ring-sensitive structure and a waveguide antenna. The flexible substrate adopts polyimide (PI) featuring low dielectric loss, high-temperature resistance and excellent flexibility, which ensures stable electromagnetic properties of the sensor under bending, twisting and curved surface attachment conditions. As the core component determining sensor performance, the resonant ring’s geometric dimensions, line width and split gap directly affect the resonant frequency, sensitivity and quality factor (Q-factor). The waveguide antenna establishes electromagnetic coupling with external test equipment to transmit microwave energy and collect echo signals. Figure 1a illustrates the working principle diagram of the wireless passive crack sensor. External test equipment radiates microwave signals of specific frequency bands into space via the waveguide antenna. When the microwave frequency matches the natural resonant frequency of the sensor’s resonant ring, intense electromagnetic resonance occurs in the resonant ring, absorbing substantial local energy and forming a distinct dip on the S11 return loss curve. Once cracks emerge on the surface of the measured structure and propagate to the sensor area, they alter the effective conductive length, equivalent inductance and equivalent capacitance of the resonant ring, thereby shifting the resonant frequency of the entire LC resonant circuit. The crack width can be inversely derived by capturing the resonant frequency offset through a vector network analyzer. An equivalent circuit model is established for analysis to clearly interpret the sensor’s working mechanism, as shown in Figure 1b. The resonant ring is equivalent to a series-resonant circuit consisting of inductance *L_S_* and capacitance *C_S_*, where the inductance is determined by the resonant ring’s geometric dimensions and the capacitance is governed by inter-ring coupling. The waveguide antenna is equivalent to a combination of transmission lines and radiation impedances to achieve efficient microwave signal transmission and spatial coupling. The resonant frequency of the sensor is expressed by Equation (1):

(1) $$f=\frac{1}{2\pi \sqrt{L_{s}C_{s}}}$$

When there are no cracks on the surface of the tested structure, the resonant ring maintains its complete geometric shape, and both *L_S_* and *C_S_* remain constant, so the resonant frequency *f* stays stable. When cracks form on the structure and propagate into the resonant ring area, the crack width *w* directly alters the effective conductive length and electric field distribution of the resonant ring, which in turn causes regular changes in the equivalent inductance *L_S_* and equivalent capacitance *C_S_*. For planar-resonant ring structures, crack propagation lengthens the current path and increases the effective electrical size of the ring, resulting in an approximately linear increase in the equivalent inductance *L_S_* with the growth of crack width *w*. Meanwhile, cracks modify the local coupling capacitance, making the equivalent capacitance *C_S_* change monotonically with crack width *w*. Within the small linear operating range, the variations in inductance and capacitance can be approximately expressed as

$$L_{S}=L_{0}+L_{m}$$

$$C_{S}=C_{0}+C_{m}$$

In the formula, *L*_0_ and *C*_0_ represent the initial inductance and capacitance when there are no cracks; $L_{m}$ and $C_{m}$ stand for the variations in equivalent inductance and capacitance induced by surface cracks, respectively. Substituting the above relationships into Formula (1), it can be concluded that the crack width *w* directly determines the offset of the resonant frequency *f*(*w*):

(2) $$f\left(w\right)=\frac{1}{2\pi \sqrt{\left(L_{0}+L_{m})(C_{0}+C_{m}\right)}}$$

From the perspective of the electromagnetic field perturbation principle, the resonant behavior of the sensor is determined by the localized electromagnetic field characteristics of the resonant ring structure. When the sensor is attached to a metal surface, the metal plate can be regarded as a Perfect Electric Conductor (PEC) boundary. The resonant ring resonates under microwave excitation, and electric and magnetic field energies are highly concentrated at the edges and gap regions of the resonant ring, forming a strong localized field. When cracks appear in the measured structure, the PEC boundary on the metal surface is damaged, the distribution of the localized field undergoes significant perturbation, the resonance conditions are directly altered, and the resonant frequency shifts. When the electromagnetic field near the resonant structure is perturbed, the relative resonant frequency shift satisfies

(3) $$\frac{∆f_{r}}{f_{r}}=\frac{\int_{v}(∆\varepsilon E_{1}\cdot E_{0}+∆\mu H_{1}\cdot H_{0})dv}{\int_{v}\left(\varepsilon_{0}{\left|E_{0}\right|}^{2}+\mu_{0}{\left|H_{0}\right|}^{2}\right)dv}$$

where *f*_r_ denotes the natural resonant frequency without cracks; $∆f_{r}$ represents the frequency shift induced by cracks; $\varepsilon_{0}$ is the permittivity without cracks; $\mu_{0}$ is the permeability without cracks; ∆ε represents the change of relative permittivity within the perturbed region; ∆μ represents the change of permeability within the perturbed region; *E*_0_ and *H*_0_ stand for the original electric and magnetic field distributions in the absence of cracks; *E*_1_ and *H*_1_ refer to the electric and magnetic field distributions perturbed by cracks; and *V* is the effective region where field intensity concentrates. For cracks on metal surfaces, the emergence of cracks is equivalent to an abrupt change in local electrical boundary conditions, which drastically alters the equivalent permittivity of this region. Given that the electric field energy of resonant rings is highly concentrated within a narrow zone above the metal surface, tiny cracks can trigger substantial field perturbations, drastically increase the molecular value, and ultimately lead to a remarkable shift in resonant frequency.

## 3. Simulation of a Wireless Passive Crack Sensor

**Figure 2:**
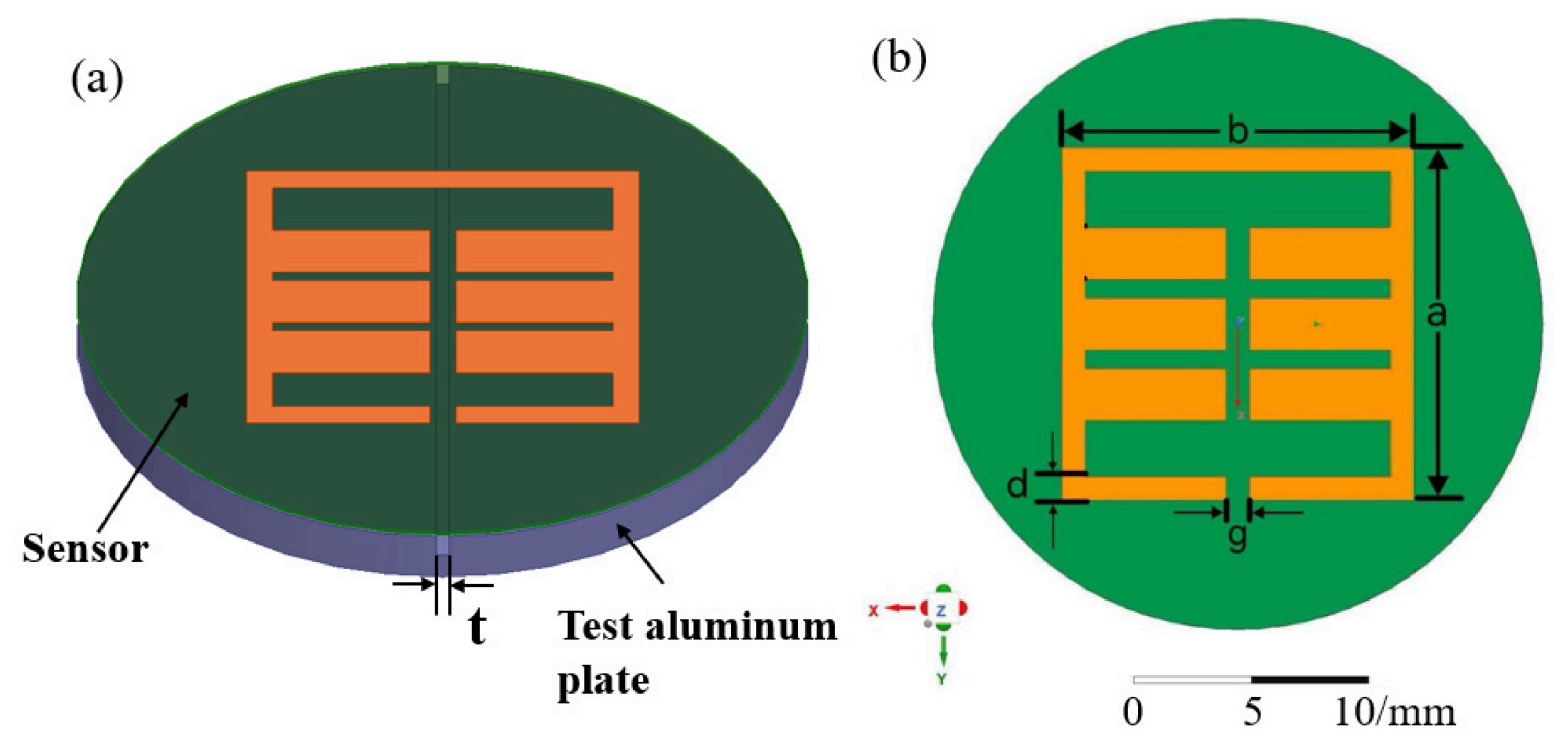
Sensor simulation model diagram. (**a**) Nondestructive crack sensor detection model. (**b**) The sensor’s dimensional parameters.

The dimensional drawing of the sensor model is shown in Figure 2. To optimize the sensor structure and improve the crack width detection performance, this paper establishes a three-dimensional model of a flexible wireless passive crack sensor using Ansys’ High-Frequency Structure Simulator (HFSS, Ansys, Canonsburg, Pennsylvania, USA 2015), as illustrated in Figure 2b, and conducts systematic simulation analysis on the resonant ring dimensions, crack width response and electric field distribution. The simulation model is built strictly in accordance with the actual structural dimensions. Key parameters including the dielectric constant and loss tangent of the flexible substrate as well as metal conductivity are configured. Radiation boundary conditions are adopted to simulate an open space, and wave port excitation is utilized to realize broadband frequency sweeping so as to obtain the S11 return loss curve, the resonant frequency, the electric field distribution and the coupling influence law of multiple factors. The sensor adopts an integrated planar structure with the resonant ring located in the central sensitive area and waveguide antennas arranged on both sides, which ensures uniform energy coupling, sharp resonant peaks and a high Q factor.

**Figure 3:**
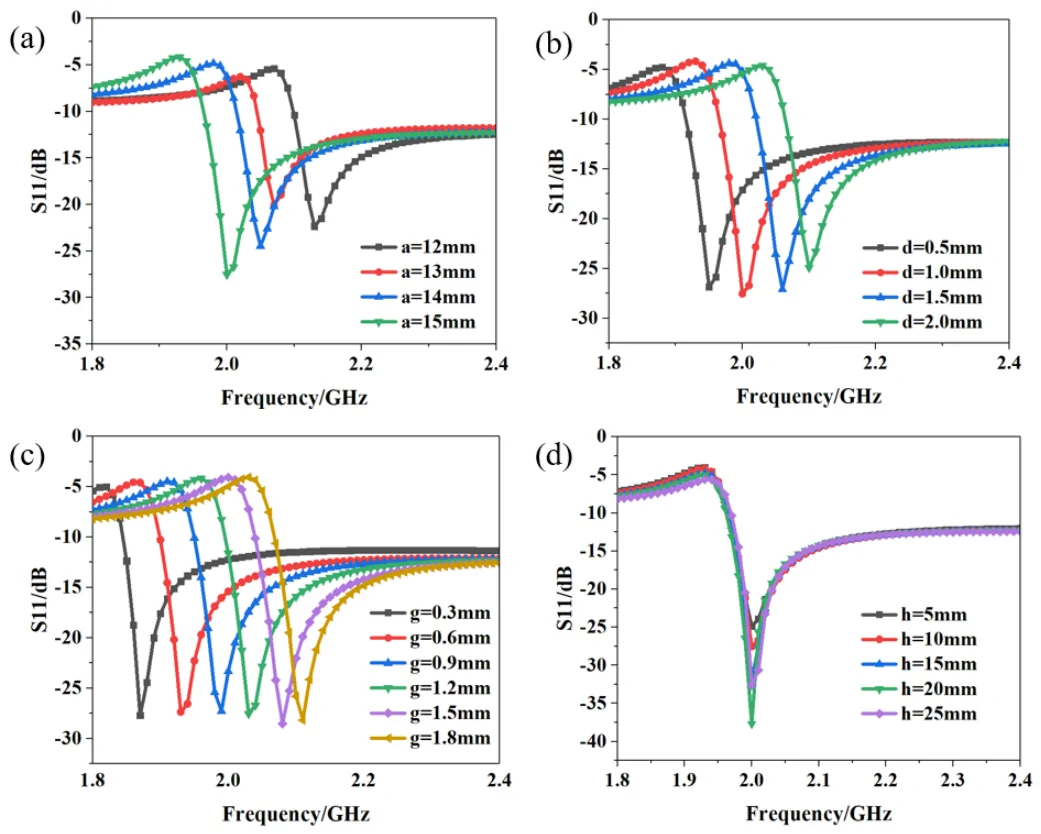
Sensor simulation model diagram. The influence of parameters: (**a**) Resonant ring length a. (**b**) Resonant ring width b. (**c**) Resonant ring split gap g. (**d**) Distance between antenna and sensor h.

Parametric scanning is performed to optimize the side length, line width, opening gap of the resonant ring, substrate thickness and antenna length, enabling the sensor to possess optimal resonant characteristics and crack width sensitivity within the target frequency band. Figure 3 presents the optimization results of the key sensor parameters. It can be seen from Figure 3a that the resonant frequency of the sensor decreases as the size of the resonant ring increases. Figure 3b shows the effect of resonant ring width on the sensor’s resonant frequency, which gradually declines with the growth of resonant ring width. Figure 3c reflects the influence of the resonant ring opening gap on the sensor’s resonant frequency; the resonant frequency of the sensor rises gradually as the opening gap increases. Figure 3d demonstrates the impact of the distance between the interrogation antennas on the sensor’s resonant frequency. As indicated in Figure 3d, the distance mainly affects the resonant intensity of the sensor and exerts a minor influence on its resonant frequency. When the distance increases from 5 mm to 20 mm, the sensor’s resonant intensity rises progressively and reaches −38 dB at a distance of 20 mm. A higher resonant intensity of the sensor corresponds to higher detection accuracy in actual tests. As the distance further increases to 25 mm, the sensor’s resonant intensity decreases gradually. Simulation results reveal that reasonable structural parameters can significantly reduce return loss, strengthen resonant peak intensity and enhance the anti-interference capability of the sensor. Joint simulation and iterative optimization of multiple parameters were carried out with the maximization of the Q-factor as the optimization objective, and the optimal parameter combination was finally determined. The corresponding parameter values are as follows: a = 15 mm, b = 1 mm, g = 1 mm, and h = 15 mm.

**Figure 4:**
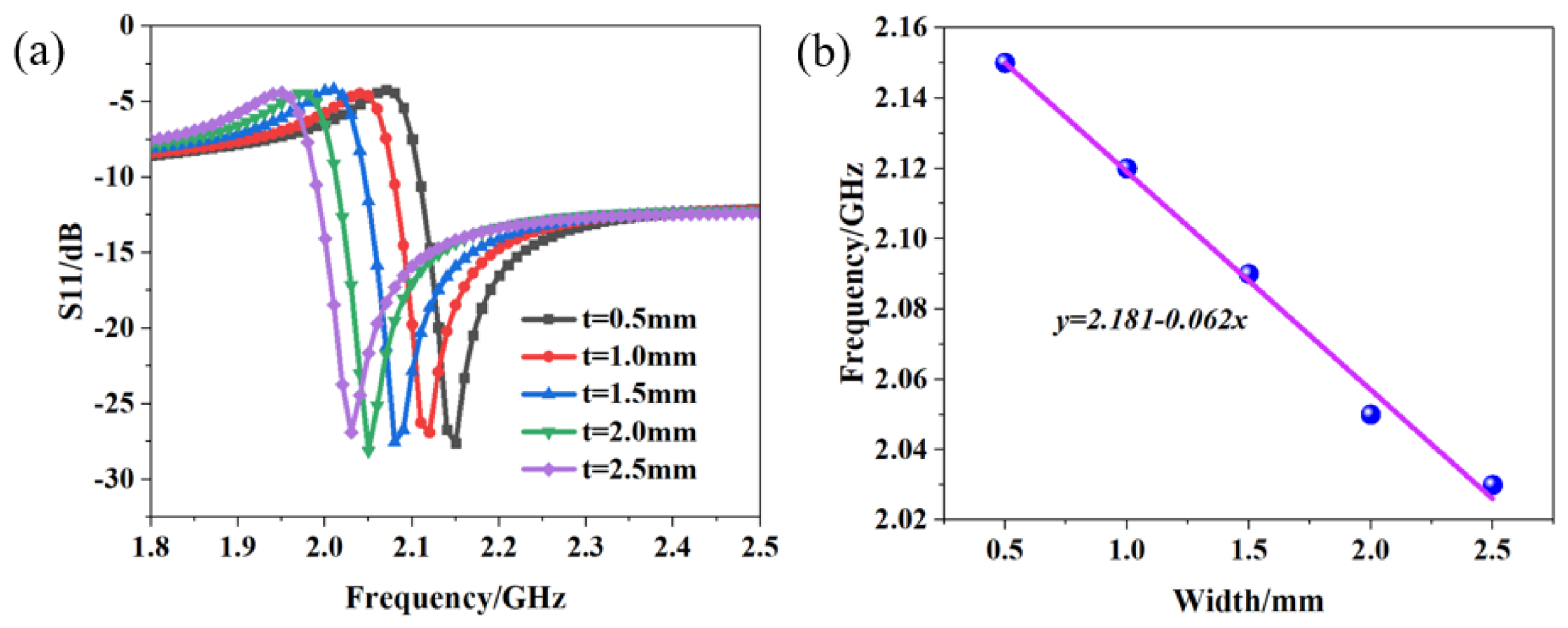
Effect of different crack widths on the resonant frequency of the sensor. (**a**) Frequency curves under various crack widths. (**b**) Fitting curve.

In the simulation of crack width sensitivity, the crack propagation process within the range of 0–2.5 mm is simulated by gradually varying the crack width, and the resonant frequency offsets under different widths are extracted. The simulation results are presented in Figure 4. The results indicate that the resonant frequency of the sensor exhibits a distinct linear decreasing trend as the crack width increases, the magnitude of return loss remains stable, and no obvious distortion occurs in the resonant peaks, which demonstrates that the sensor possesses favorable responsiveness to crack width.

**Figure 5:**
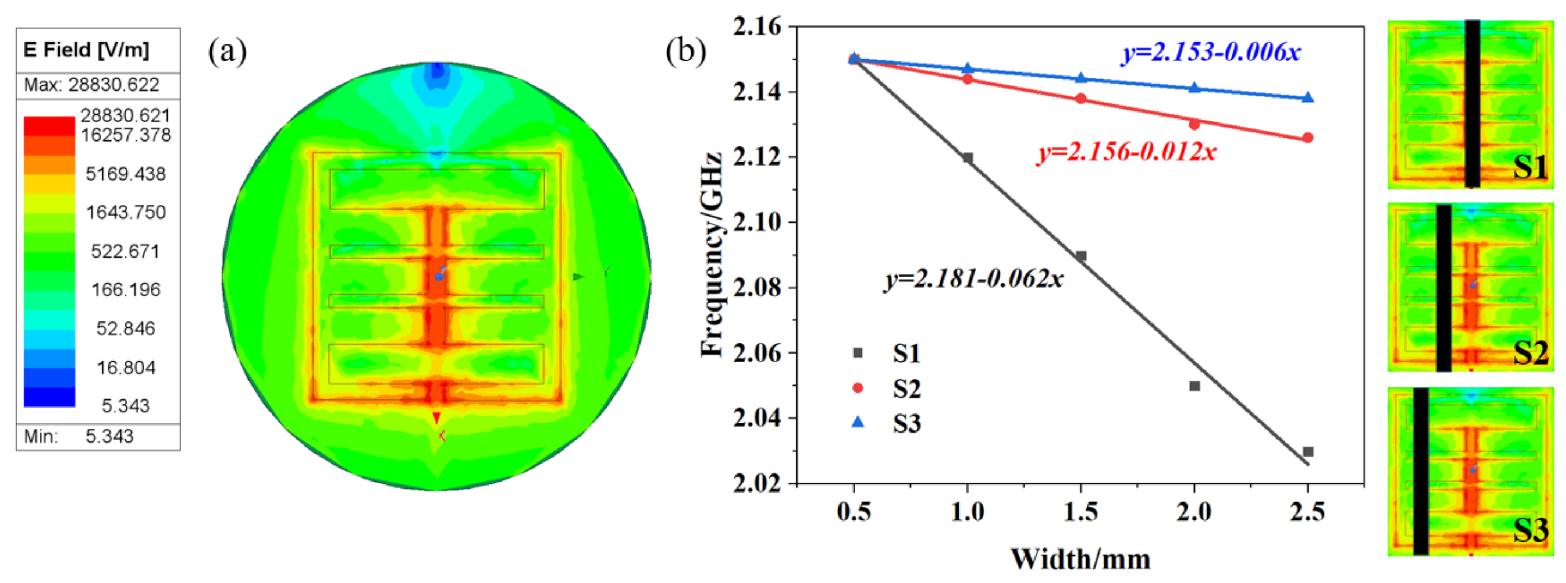
(**a**) Electric field distribution diagram of the sensor. (**b**) The effect of crack position on sensor sensitivity.

To further reveal the sensing mechanism, electric field distribution simulation is carried out for the resonant region, as shown in Figure 5a. The results indicate that electric field energy is highly concentrated at the split gap of the resonant ring. Crack propagation will directly damage the electric field concentration area and significantly alter the resonant state, thereby improving detection sensitivity. Figure 5b investigates the effect of crack position on sensor detection sensitivity, where the horizontal axis represents crack width ranging from 0.5 mm to 2.5 mm and the vertical axis corresponds to the resonant frequency of the sensor. The black rectangular strips in the electric field cloud images on the right denote crack positions: S1 refers to the crack located in the region of maximum electric field intensity, S2 stands for a moderate offset from the strong-field zone, and S3 represents a large offset. Linear fitting is performed for all three groups of test data. The fitting equation of S1 is *y* = 2.181 − 0.062*x*, which has the largest absolute slope value, leading to the most obvious resonant frequency drop with increasing crack width and the highest detection sensitivity. By contrast, the fitting formulas of S2 (*y* = 2.156 − 0.012*x*) and S3 (*y* = 2.153 − 0.006*x*) possess successively smaller absolute slopes, resulting in milder frequency variation and gradually degraded sensitivity. These results clearly demonstrate that the farther the crack deviates from the sensor’s maximum electric field area, the weaker its disturbance to the electromagnetic field of the device and the lower the crack detection sensitivity; optimal sensing performance can only be achieved when the crack is placed within the peak electric field region of the interdigital gaps.

## 4. Processing and Testing of the Wireless Passive Crack Sensor

In this paper, a combined process of flexible printing and laser etching is adopted to fabricate samples of flexible wireless passive crack sensors. The entire process is carried out in a clean environment to guarantee structural precision and consistent performance. First, the flexible substrate undergoes cleaning, drying and plasma activation treatment to eliminate surface oil stains and impurities and enhance the adhesion of the metal layer. Next, high-precision screen printing or magnetron sputtering is utilized to deposit conductive metal circuits on the substrate surface, forming resonant ring-sensitive structures and waveguide antennas. Fine-pattern correction is implemented via laser etching to ensure that the dimensions of the resonant rings, opening gaps and antenna structures meet the designed precision requirements. Finally, a protective layer is coated to improve the sensor’s resistance to abrasion, moisture and aging and extend its service life. The fabricated sensors feature thinness, light weight, softness and bendability and can withstand bending with a small radius, making them suitable for conformal mounting on various curved structures.

**Figure 6:**
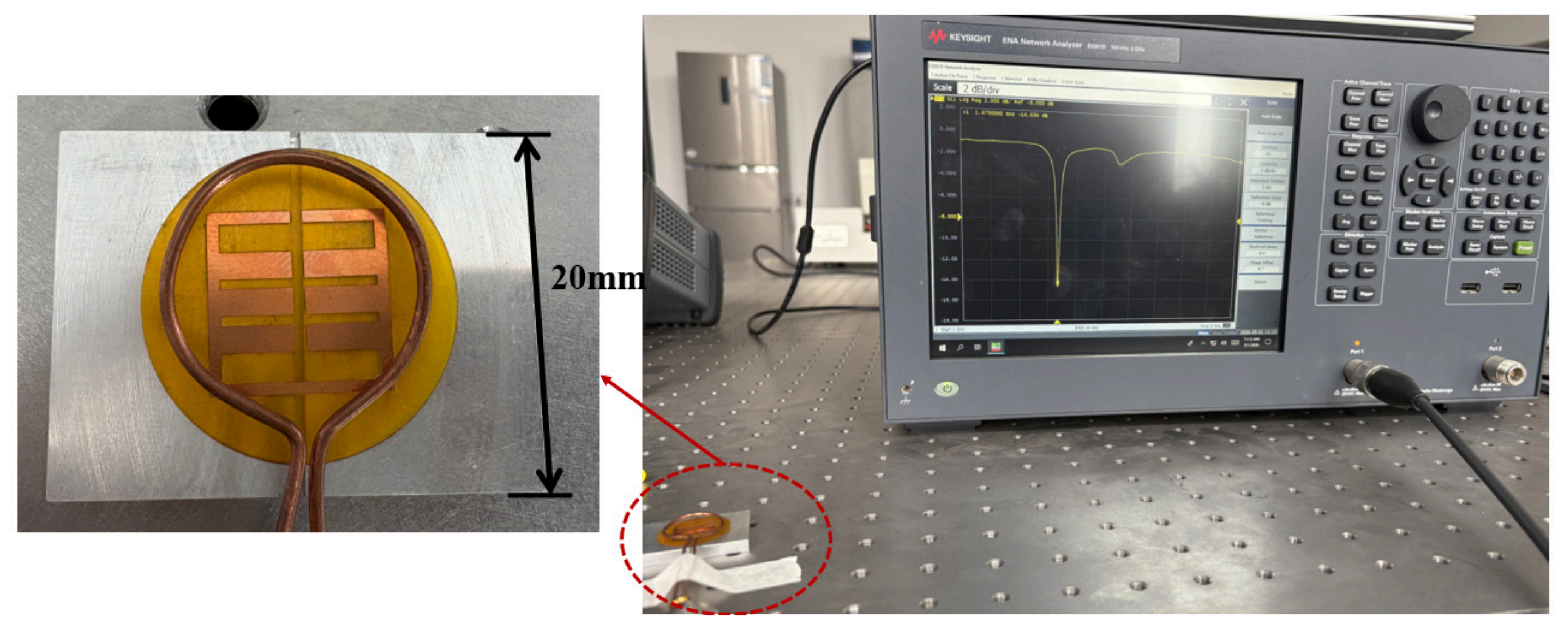
Crack sensor test platform.

The wireless passive crack detection system is illustrated in Figure 6. The complete setup consists of a vector network analyzer (Keysight, Santa Clara, CA, USA N5061B), resonant sensors, coupling coil antennas and an aluminum test plate with prefabricated cracks. The coil antenna is connected to the vector network analyzer via cables and transmits swept-frequency signals outward. When the frequency of the swept signal matches the inherent resonant frequency of the sensor, electromagnetic waves are coupled into the interior of the sensor and generate reflections, and the reflected signals are transmitted back to the vector network analyzer through the coil antenna. Accordingly, the response curve collected by the vector network analyzer represents the resonant characteristic curve of the sensor, and the frequency corresponding to the valley of the curve is the resonant frequency of the sensor.

**Figure 7:**
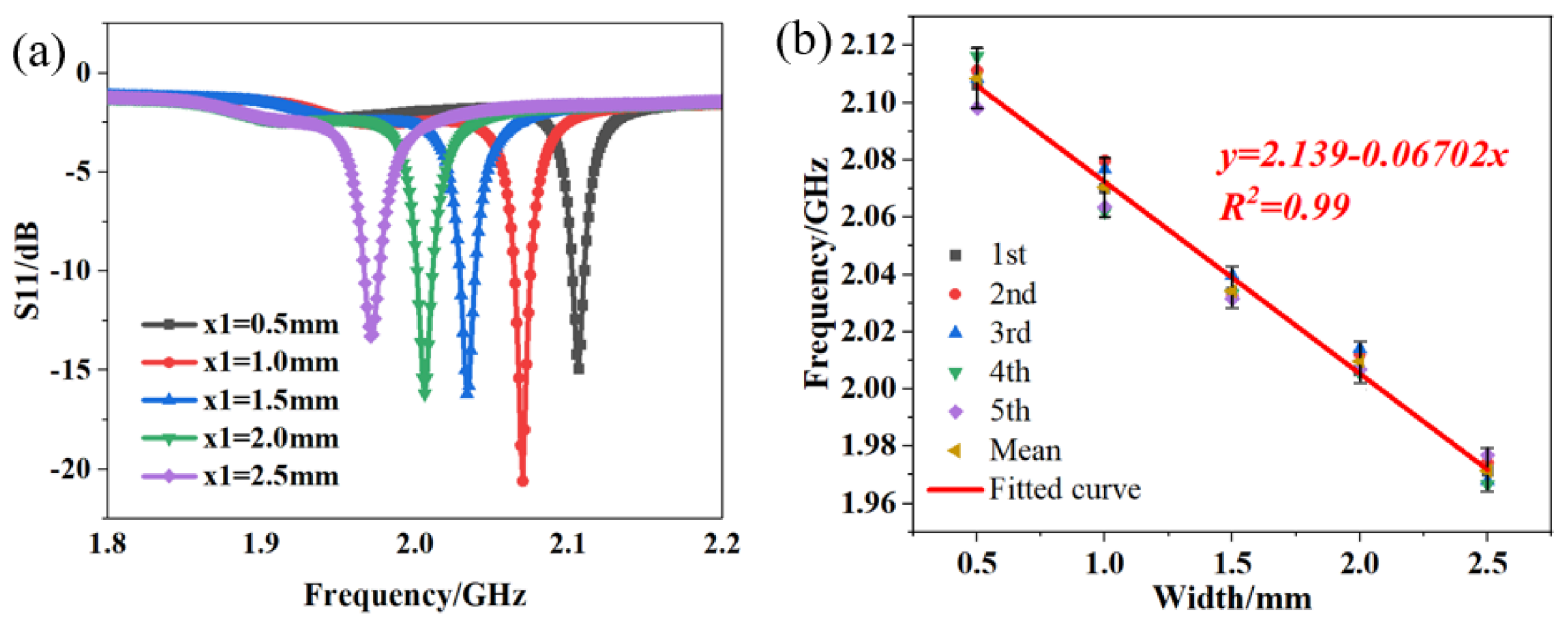
Sensor test results and analysis. (**a**) Frequency curves under various crack widths. (**b**) Analysis of multiple measurements.

The test contents mainly consist of three parts: a static crack width response test, a multi-environment influence test and a flexibility adaptability test. In the static calibration experiment, the gap widths of the tested aluminum plates are 0.5 mm, 1 mm, 1.5 mm, 2 mm and 2.5 mm. After maintaining stability at each width point, the S11 return loss curves are recorded, the resonant frequencies are extracted, and a calibration curve of crack width versus resonant frequency is established, as shown in Figure 7a. Figure 7b shows the repeatability test results of the sensor for crack width detection. Five repeated measurements were carried out at crack widths of 0.5 mm, 1.0 mm, 1.5 mm, 2.0 mm and 2.5 mm, which are marked as the first to fifth test data points with different colored symbols. The vertical error bars at each data point represent the frequency fluctuation range of repeated measurements, and the yellow triangular points denote the average resonant frequency of five replicates under the same crack width. A linear regression fitting is performed on the average data, with the fitted curve drawn in red. The fitting equation is *y* = 2.139 − 0.06702*x*, with the coefficient of determination R^2^ = 0.99. Small error bars across all test conditions demonstrate that the sensor possesses outstanding measurement repeatability and stability for crack width quantification. The experimental results indicate that the sensor exhibits an excellent linear relationship within the full measurement range; the resonant frequency decreases steadily as the crack width increases, with a sensitivity of 67.02 MHz/mm, which meets the requirements of engineering detection. The resonant peaks are distinct without clutter interference, and the consistency of the repeated tests is favorable.

**Figure 8:**
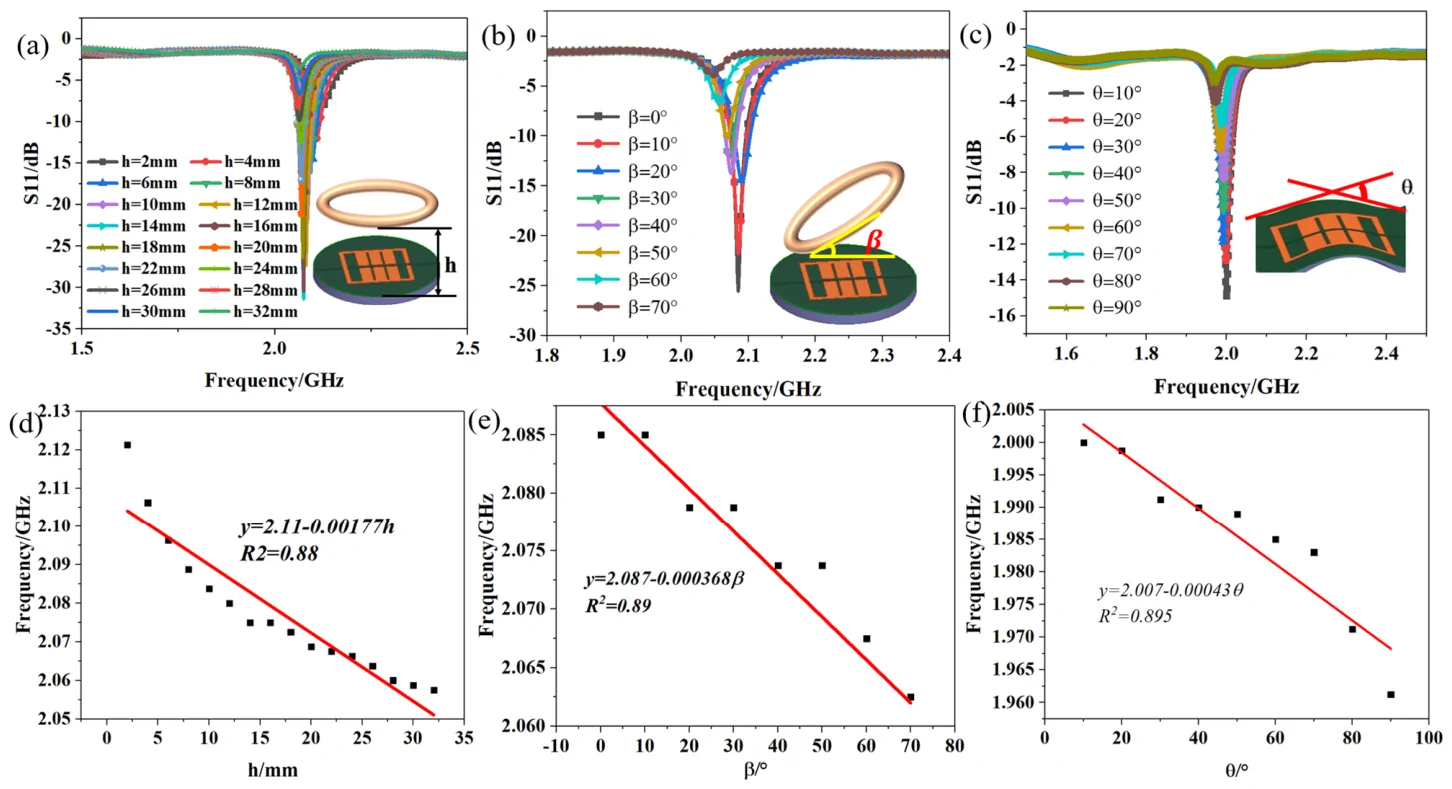
Effects of (**a**) distance, (**b**) included angle and (**c**) curvature on the resonant frequency of the sensor. (**d**) Resonant frequency versus antenna–sensor spacing, *h*. (**e**) Resonant frequency versus antenna–sensor angle, *β*. (**f**) Resonant frequency versus sensor bending angle, *θ*.

In environmental impact experiments, the distance between the sensor and the coil antenna, the angle between the sensor and the interrogation antenna, and the substrate bending curvature were adjusted separately, and the changes in output characteristics were recorded, as shown in Figure 8. Figure 8a illustrates the effect of the distance between the sensor and the coil antenna on the resonant frequency of the sensor. It can be seen that within a reasonable operating distance, the frequency drift of the sensor is slight, enabling stable wireless reading performance. Figure 8b presents the output curve of the sensor under varying angles relative to the interrogation antenna. The results reveal that small angular deflections exert little influence on detection outcomes, offering a high tolerance for practical installation. Figure 8c depicts the impact of substrate bending curvature on the sensor’s resonant frequency. The sensor maintains stable output under bending conditions without obvious degradation of resonant characteristics, verifying its outstanding flexible conformability. To further quantify the influence of the three environmental factors on resonant frequency, the resonant frequency points corresponding to each test condition are extracted from the S_11_ spectra, and linear regression fitting is performed for each variable, as shown in Figure 8d–f. Figure 8d plots the linear relationship between resonant frequency and antenna–sensor separation distance, with the fitting equation *f* = 2.11 − 0.00177*h* and the coefficient of determination R^2^ = 0.88. The fitting slope of −0.00177 GHz/mm quantitatively characterizes the frequency shift sensitivity induced by reading distance variation, confirming the mild frequency drift within the effective operating range. Figure 8e displays the linear fitting result of resonant frequency versus antenna tilt angle, yielding the regression formula *f* = 2.087 − 0.000368*β* (R^2^ = 0.89). The relatively small absolute slope indicates that angular misalignment introduces limited frequency deviation, which is consistent with the high installation tolerance observed in the S_11_ curves. Figure 8f presents the linear fitting of resonant frequency against substrate bending angle, as described by *f* = 2.007 − 0.00043θ with R^2^ = 0.895, demonstrating that bending deformation causes a nearly linear monotonic decrease in resonant frequency while maintaining good linear correlation. By comparing the absolute values of the three fitting slopes, it can be concluded that antenna separation distance exerts the most significant modulation effect on resonant frequency, followed by substrate bending curvature, while antenna tilt angle has the weakest influence. These quantitative fitting results not only provide explicit sensitivity indicators for each environmental factor but also establish a mathematical basis for subsequent frequency drift compensation and crack width inversion under complex application conditions. Experimental test results indicate that the proposed flexible wireless passive crack sensor exhibits decent sensitivity and acceptable linearity within the tested crack width range, along with flexible deformability, a wireless passive working mode and easy installation. It is capable of realizing non-contact monitoring of structural crack widths under laboratory conditions, which shows potential for long-term stable measurement in practical engineering applications.

To objectively evaluate the comprehensive performance of the sensor proposed in this paper, ten representative high-quality papers published in recent years are selected for comparison of six key indicators, namely structural type, power supply mode, flexibility, sensitivity, readout method and substrate compatibility, as shown in Table 1:

It can be seen from Table 1 that most existing similar sensors adopt rigid structures with poor flexible adaptability, making conformal installation on curved surfaces difficult. Although some sensors achieve wireless and passive operation, they suffer from limited detection ranges, insufficient linearity, or high sensitivity to environmental factors. The sensor designed in this paper realizes dedicated, stable and high-precision monitoring of crack width. It boasts prominent advantages in flexible conformal performance, wide-range linear responses, compatibility with multiple base materials and environmental stability while retaining features such as high sensitivity, a wireless passive design, a simple structure and low costs, making it more suitable for long-term distributed deployment at engineering sites.

## 5. Conclusions

This paper successfully develops a flexible wireless passive crack sensor based on resonant rings, completing full-process research covering working mechanism analysis, structural design, electromagnetic simulation, fabrication, and system testing and realizing high-sensitivity, wireless, passive, flexible and conformal monitoring of structural crack widths. Using a flexible polymer as the substrate, the sensor integrates resonant ring-sensitive structures and waveguide antennas into a single unit. It modulates resonant parameters with the expansion of crack widths and achieves non-contact readout via microwave echo signals, featuring prominent advantages such as battery-free operation, no wired connections, high stability and a long service life. Simulation and experimental results demonstrate that the sensor delivers an excellent linear response within the crack width range of 0–2.5 mm; the resonant frequency decreases monotonically with the increase in crack width and is accompanied by high sensitivity and favorable repeatability. It maintains stable output under varying test distances, mounting angles and bending curvatures, exhibiting strong environmental adaptability and outstanding flexible conformal performance. The sensor proposed in this paper boasts comprehensive advantages in flexible adaptability, detection linearity, multi-substrate compatibility and engineering practicability. This sensor can be widely applied to long-term online monitoring of crack widths in infrastructures and equipment including bridges, tunnels, rail transit, aerospace structures, wind turbine blades, pressure pipelines and new energy facilities, enabling early crack identification, propagation trend prediction and structural safety assessment. Future research can further explore miniaturized designs, multi-sensor array networking, extended wireless readout distances and intelligent demodulation algorithms, thus advancing flexible wireless passive crack width sensing technology toward distributed, intelligent and networked development and providing more reliable, efficient and low-cost innovative technical support for structural health monitoring of major engineering projects.

## Figures and Tables

**Table 1:** Comparison of performance parameters of different crack sensing schemes.

Ref.	Sensor Structure	Power Supply	Flexibility	Sensitivity (MHz/mm)	Range (mm)	Test Object
[13]	WiFi Sensor	Active	No	116	0–5	Metal
[15]	Microwave Resonator	Active	No	--	--	Rock/Concrete
[20]	Chipless Hybrid RFID	Wireless Passive	No	31–117	1–5	Metal
[21]	Passive RFID Sensor	Wireless Passive	No	114–128	1.5–2.5	Metal
[22]	Microwave Sensor	Passive	No	63.3–100	1.5–2.5	Metal
[24]	Flexible Sensor Tag	Wireless Passive	Yes	43	Non-contact	Metal/Composite
[25]	Tap Sensor	wireless Active	No	--	0–2.5	Metal
This work	Flexible Resonant Loop Sensor	Wireless Passive	Yes	67.02	Non-contact	Metal

## Data Availability

The data presented in this study are available in this article.
